# Supplementary material for: The Impact of the Anticoagulant Type in Blood Collection Tubes on Circulating Extracellular Plasma MicroRNA Profiles Revealed by Small RNA Sequencing
Source: Int J Mol Sci. 2022 Sep 7;23(18):10340. doi: 10.3390/ijms231810340 (PMC9499385; doi:10.3390/ijms231810340)
Supplement: Supplementary file 1 [file ijms-23-10340-s001.zip › Supplementary Figures.pdf]

Supplementary Figures

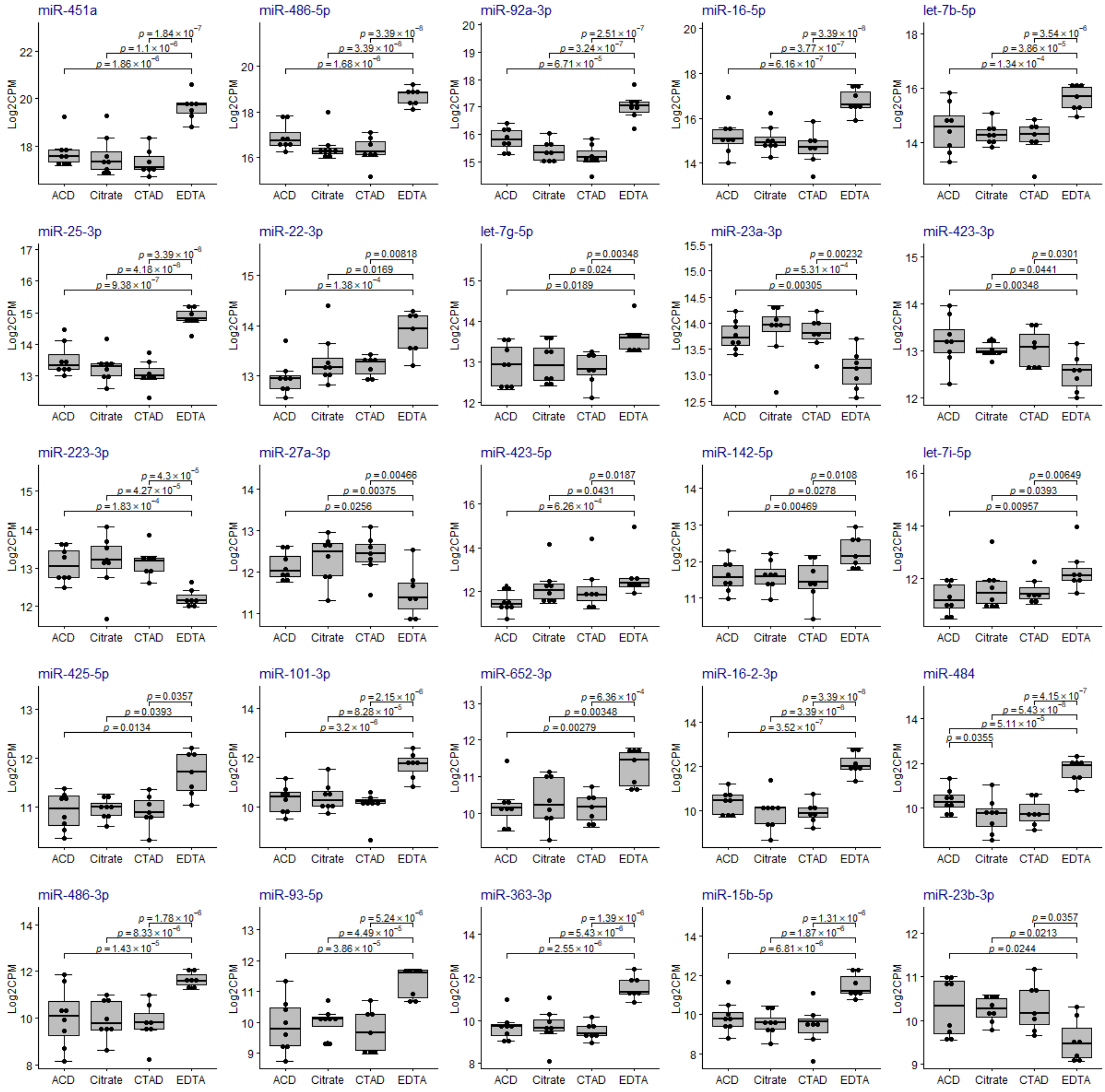

Figure S1. The distribution of miRNA expression for the differentially expressed miRNAs in the study sample groups (part 1). On the y-axis, TMM-normalized log2CPM values are shown. The boxplots represent median and interquartile ranges (IQRs) in the box, and values for individual samples in the dots. *P*-values of statistical significance for the pairwise quasi-likelihood F-test comparisons performed in EdgeR are provided on the plots for each pair of the study groups with $p < 0.05$.

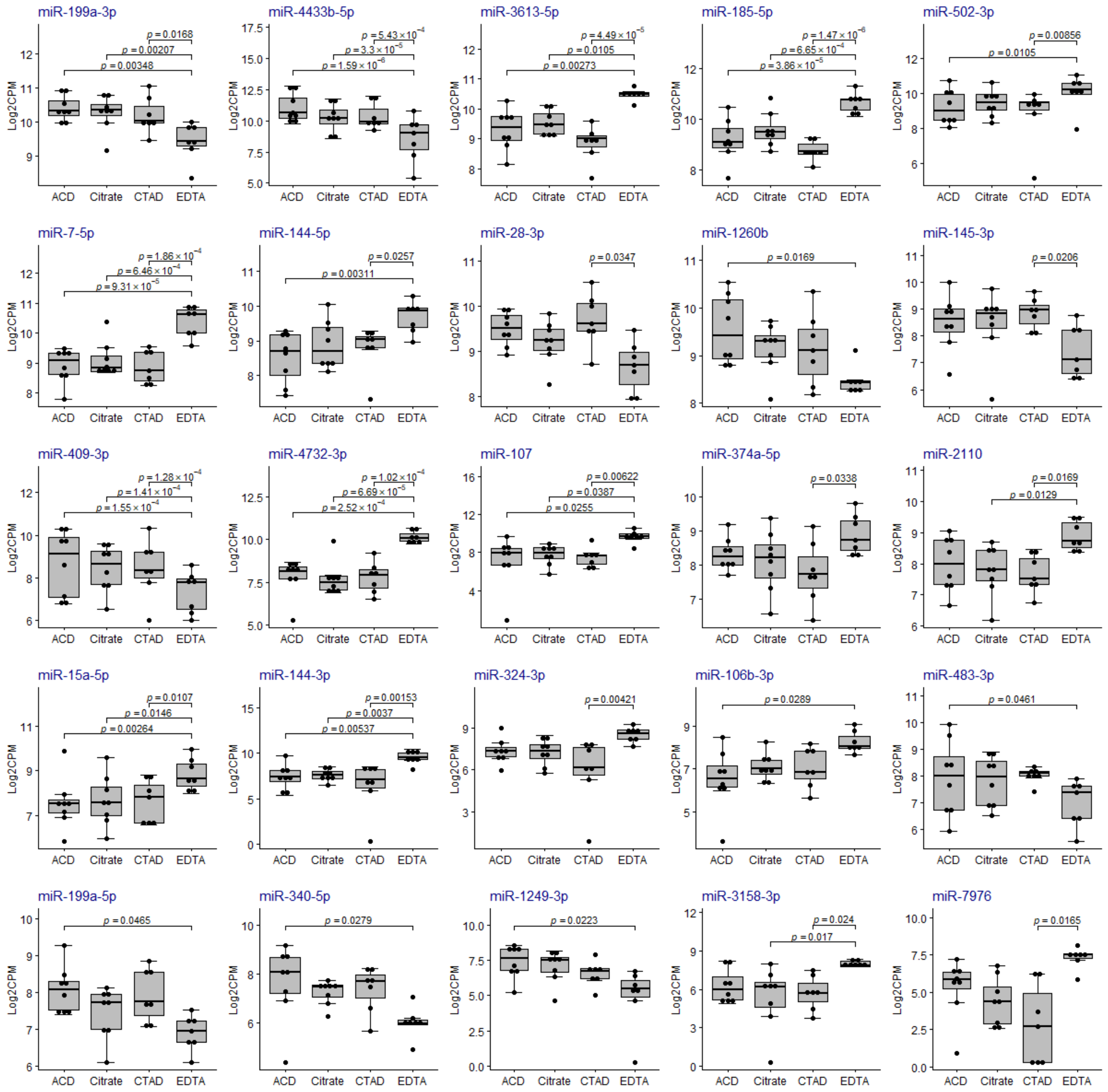

Figure S2. The distribution of miRNA expression for the differentially expressed miRNAs in the study sample groups (part 2). On the y-axis, TMM-normalized log2CPM values are shown. The boxplots represent median and interquartile ranges (IQRs) in the box, and values for individual samples in the dots. *P*-values of statistical significance for the pairwise quasi-likelihood F-test comparisons performed in EdgeR are provided on the plots for each pair of the study groups with $p < 0.05$.

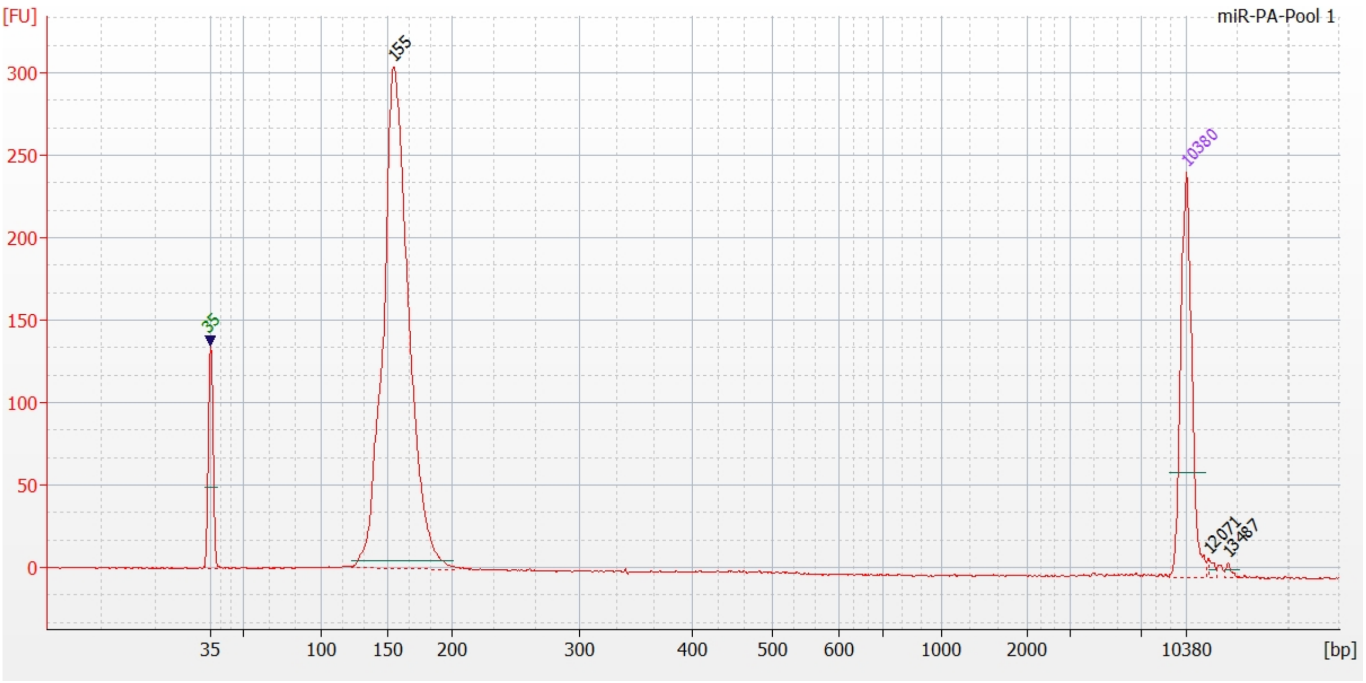

Figure S3. The size distribution of the miRNA library pool on a Bioanalyzer 2100 electrophoregram.

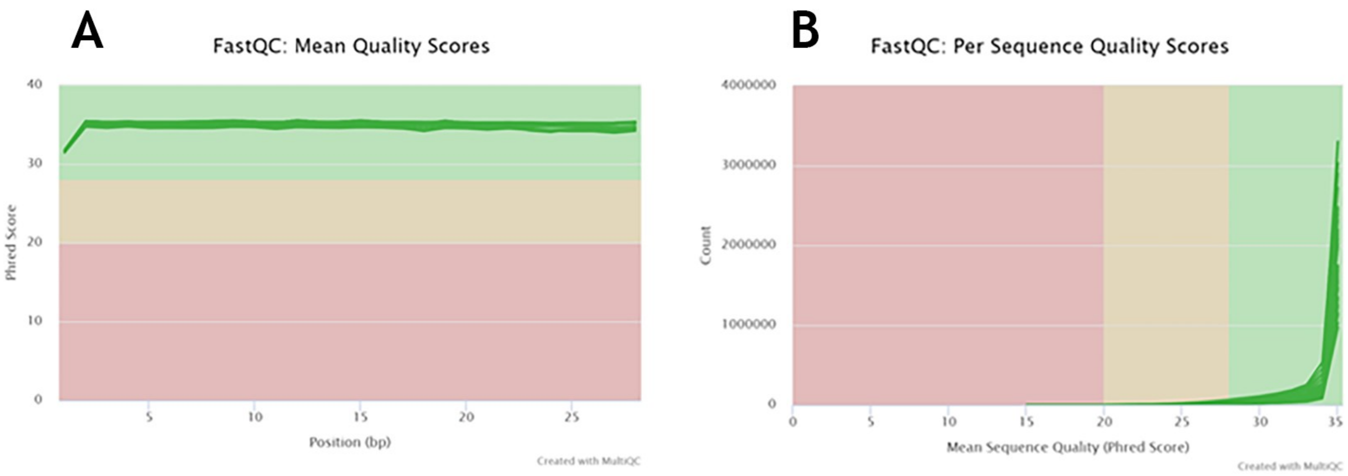

Figure S4. Visualization of the sequencing read quality using the FastQC program tool in the pool of reads filtered for miRNA analysis. (A) Mean quality scores (Phred score) depending on the position in the sequencing read. (B) Distribution of read count depending on the mean quality score (Phred score).
